# Minimal Residual Disease Detection: Bridging Molecular and Clinical Strategies for Recurrence Prevention in Gynecologic Cancers

**DOI:** 10.3390/ijms262311708

**Published:** 2025-12-03

**Authors:** Andi Darma Putra, Naufal Syafiq Darmawan, Aldi Tamara Rahman, Lasmini Syariatin

**Affiliations:** 1 Division of Gynecology-Oncology, Department of Obstetrics and Gynecology, Faculty of Medicine, Universitas Indonesia, Cipto Mangunkusumo Hospital, Central Jakarta 10430, Indonesia; 2 Dopamine Science Institute, Pancoran Mas, Depok 16431, Indonesia; 3 Assistant Division of Gynecology-Oncology, Department of Obstetrics and Gynecology, Cipto Mangunkusumo Hospital, Central Jakarta 10430, Indonesia

**Keywords:** gynecologic cancer, cancer detection, minimal residual disease, cancer recurrence, circulating tumor DNA, circulating tumor cells

## Abstract

Gynecologic cancers remain a major global health burden, particularly in low- and middle-income countries, with high incidence and mortality rates around 45–50%. The detection of minimal residual disease (MRD) is transforming the management of recurrence risk in gynecologic cancers through highly sensitive molecular technologies. MRD encompasses small populations of residual cancer cells or post-treatment molecular traces but remain undetectable by conventional methods. Its detection relies on circulating tumor DNA (ctDNA), circulating tumor cells (CTCs), and advanced next-generation sequencing (NGS), with ctDNA-based MRD assays having sensitivity levels between 85% and over 99%. Other technologies, such as liquid biopsies and digital PCR, are also in development. MRD status has demonstrated high predictors of recurrence and survival with positive MRD strongly associated with poor outcomes and negative MRD indicates sustained remission. However, MRD detection faces significant limitations, such as tumor heterogeneity, inconstant ctDNA levels, technical issues of false-negative results, and limited clinical accessibility. Therefore, this review presents current evidence regarding the molecular detection of MRD in gynecologic malignancies and assesses its prognostic and predictive relevance. Ultimately, MRD continuous integration into clinical practice offers a promising modality to enable early relapse detection, more precise therapeutic decision-making, and the improvement of personalized medicine access to gynecologic cancers worldwide.

## 1. Introduction

Gynecologic cancers remain a leading cause of global mortality, especially in low- and middle-income countries. In 2022, an estimated 1,473,427 new cases were reported, with cervical cancer representing the largest incidence (44.95%). In the same year, gynecologic cancers resulted in 680,372 deaths, with cervical cancer contributing the highest percentage (51.28%) [1]. The greatest incidence and mortality rates were observed in low- and middle-income countries among both premenopausal and postmenopausal women, particularly in East Asia and Southern sub-Saharan Africa [2]. Despite the significant global burden, available diagnostic techniques still have problems in accurately identifying early or recurrent diseases, highlighting the importance for better diagnostic precision. Advances in cancer therapies and surveillance systems have reduced incidence rates and improved long-term survival. For ovarian cancer, CA125 screening and transvaginal ultrasound remain the most frequently used diagnostic tools, often combined with complementary approaches such as the Risk of Ovarian Malignancy Algorithm (ROMA) to enhance diagnostic accuracy up to 91.2% [3]. For cervical and endometrial cancers, human papillomavirus (HPV) testing, Papanicolaou (Pap) smear cytology, polymerase chain reaction (PCR), and biopsy are widely applied, reaching sensitivity levels as high as 99% [4,5]. However, diagnostic performance may vary depending on factors such as age, International Federation of Gynecology and Obstetrics (FIGO) stage, tumor size, histopathological type, and lymph node metastasis. In treatment, surgical staging with primary debulking surgery and lymph node assessment, combined with radio-chemotherapy remains the standard strategy associated with a lower risk of metastasis.

Despite these advances, significant challenges remain in recognizing early recurrence and maintaining clinical remission. These limitations may delay diagnosis, limit therapeutic options, and ultimately decrease survival rates. The application of CA125 is limited by false positives and poor correlation with recurrence, with diagnostic accuracy of only 13% [6,7]. In addition, conventional imaging methods, including computed tomography (CT), positron emission tomography (PET), magnetic resonance imaging (MRI), and ultrasonography (USG), are also ineffective in identifying microscopic residual disease, particularly lesions smaller than 5 mm, and are unable to identify tumor heterogeneity [8,9,10].

**Figure 1:**
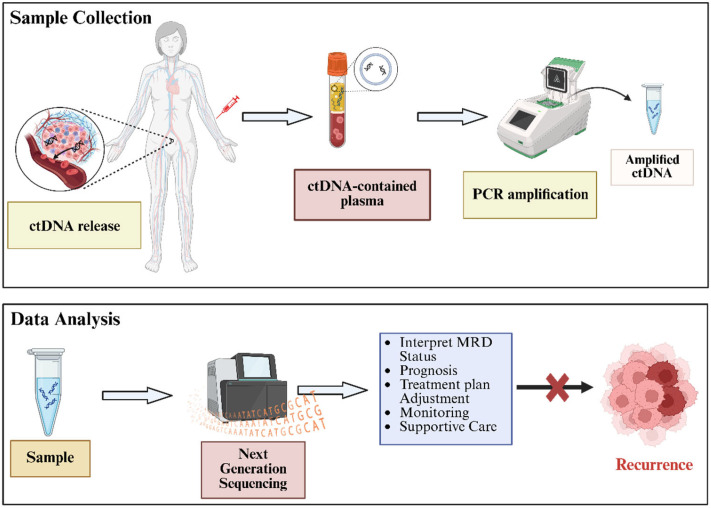
Main procedure for ctDNA-based MRD diagnosis in gynecologic malignancies. The MRD analysis results can support the early detection and prevention of cancer recurrence.

To address these limitations, minimal residual disease (MRD) monitoring has emerged as a significant advancement in oncology. MRD refers to a small population of residual cancer cells that persist post-treatment, often undetectable by conventional methods. Current MRD detection strategies include flow cytometry, PCR, next-generation sequencing (NGS), and liquid biopsy. with the latter has become the most widely used approach due to its minimally invasive properties, low risk of adverse effects, and ability to recognize tumor heterogeneity (Figure 1). Liquid biopsy relies on ctDNA, which consists of tumor-derived DNA fragments released into the bloodstream through apoptosis or necrosis [11]. This approach facilitates the identification of smaller tumor residues at the molecular level for early intervention and is fundamentally revolutionizing therapy paradigms in cancer diagnostics. Previous studies, such as those by Alimirzaie et al. (2019) have described the molecular components and clinical procedures of liquid biopsy-based MRD detection in breast cancer [12]. Further, Vacante et al. (2020) have discussed its clinical application and regulatory considerations in colorectal cancer management [13]. Although MRD screening is commonly utilized in hematologic malignancies and increasingly applied in several types of solid cancers, its implementation in gynecologic cancers remains relatively unexplored, highlighting the need for further study. This review aims to provide an in-depth summary of current advances of MRD in cancer, with emphasis on its prognostic and predictive significance in gynecologic cancers, highlighting its role in enhancing diagnostic precision, personalizing treatments, and enabling early intervention.

## 2. The Principles of Minimal Residual Disease (MRD)

### 2.1. The Evolution of MRD Detection in Oncology

Minimal Residual Disease (MRD) refers to the persistence of a small number of cancer cells, often in the form of DNA fragments or proteins, that remain in the patient’s body during or after treatment despite clinical remission. These residual cells are undetectable by conventional medical imaging, routine screening techniques, or standard histopathological analysis. The concept of MRD was initially introduced in the context of acute lymphoblastic leukemia and acute myeloid leukemia [14]. Over the past decade, with advances in sensitive detection technologies, MRD research has expanded its scope beyond hematologic malignancies to solid tumors, such as breast, colorectal, lung, ovarian, and other cancer types, signifying its growing prognostic importance. While hematological cancer predominantly depends on multiparameter flow cytometry (MPFC) and real-time quantitative polymerase chain reaction (RT-qPCR) for MRD detection, solid tumors employ different approaches due to their distinct biological properties, including differences in cellular distribution, genetic characteristics, and microenvironment [15]. In this context, ctDNA analysis via liquid biopsy has emerged as the primary modality through next-generation sequencing (NGS)-based evaluation for comprehensive tumor-specific mutations [16]. Thus, this approach could offer insights into patient survival outcomes while assisting clinicians in evaluating tumor stage and therapy response.

The application of MRD testing to solid tumors, particularly gynecologic cancers, signifies a major advancement in precision oncology. Clinical evidence has demonstrated that MRD provides higher accuracy in predicting tumor recurrence compared to conventional biomarkers or imaging technologies. This method enables earlier and more sensitive detection than common biomarkers such as CA125 and histopathology, while also offering the ability to monitor dynamic tumor behavior post-treatment. Beyond tumor recurrence monitoring, MRD allows insight into post-treatment mutations and tumor microenvironment dynamics, including anti-inflammatory activity and reduced metastasis [17,18]. This development has provided personalized tumor diagnostics utilizing whole genome sequencing and molecular barcoding approaches for each DNA molecule with remarkable sensitivity.

### 2.2. Biological Principles of MRD

**Figure 2:**
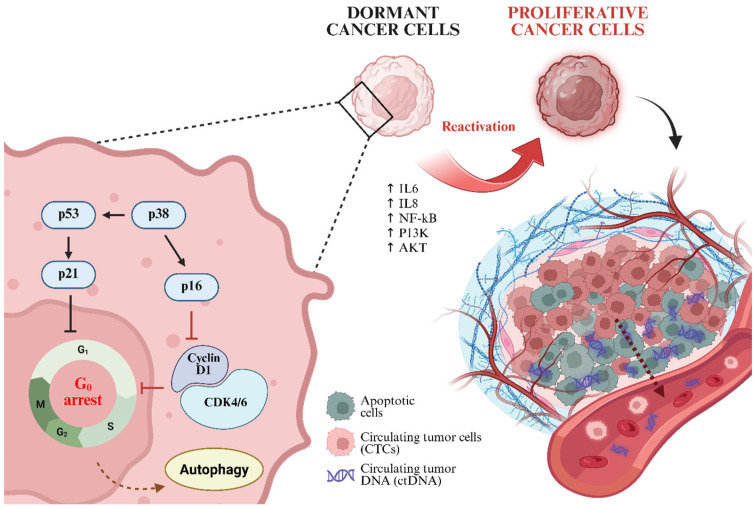
Mechanistic insight of tumor cell dormancy and reactivation, which subsequently promotes the release of ctDNA and CTCs into the bloodstream.

Residual cancer cells exhibit the remarkable ability to remain in a dormant state for prolonged periods, ranging from months to decades, before reactivation and subsequent cancer recurrence [19]. Dormancy is primarily characterized by G_0_ cell cycle arrest, during which cells enter a quiescent state and suppress their metabolic activity by reducing energy consumption, thereby enhancing their adaptability to changing microenvironment. A key regulator of this state is the activation of p8 mitogen-activated protein kinase (p38 MAPK), which further induces tumor suppressor signaling through p53 and p16. These activations subsequently downregulate extracellular signal-regulated kinase ½ (ERK1/2) and cyclin D1 to reduce proliferative signals (Figure 2) [20]. To maintain survival and the ability for reactivation, dormant cells also inactivate their P13K/AKT pathway, activate autophagy, and use stress hormones, including epinephrine, norepinephrine, and cortisol [21,22].

Other than stress-mediated survival, inflammatory conditions significantly contribute to the reawakening of dormant cancer cells. Pro-inflammatory cytokines, particularly IL-6 and IL-8, promote microenvironment susceptible to relapse. IL-6 initiates a signaling cascade that stimulates NF-kB and MAPK/ERK pathways, which further activate proliferation gene expression and facilitate G1/S cell cycle transition [23]. Moreover, changes in the immune microenvironment can relieve dormancy constraints via immune editing. While innate immune system, including NK cells, dendritic cells, macrophages, and tumor-specific T cells, play pivotal roles in eliminating residual tumor cells, persistent MRD may disrupt immune surveillance by depleting CD8^+^ T cells and upregulating the immunosuppressive programmed cell death-ligand 1 (PD-L1), thereby promoting immune evasion and recurrence [24,25,26].

In addition to cancer cell dormancy, MRD influences tumor evolution and drug resistance. Residual cancer cells may acquire sub-clonal mutations that alter treatment response compared to the primary tumor. These mutations could arise as preexisting mutations subjected to Darwinian selection or as part of adaptive evolution induced by prolonged chemotherapy exposure, representing a Lamarckian mechanism of resistance. The latter is frequently associated with the accumulation of multiple genetic and epigenetic alterations [27,28]. Drug resistance in MRD is further mediated by overexpression of ATP-binding cassette (ABC) transporters, consisting of P-glycoprotein, multidrug resistance protein 1 (MRP1), and breast cancer resistance protein (BCRP), which actively efflux chemotherapeutic drugs and reduce treatment efficacy [29]. In parallel, the release of ctDNA from residual tumor cells provides an overview of these processes. ctDNA enters the bloodstream through apoptotic and necrotic cellular turnover of tumor cells, although active secretion by surviving cells has also been demonstrated. The concentration and biological properties of ctDNA are regulated by several factors, including tumor burden, proliferation activity, and vascularization [30].

Integrating knowledge of MRD mechanisms with the resistance pathway provides opportunities to develop rational interventions that target multiple mechanisms simultaneously, thereby preventing resistance development enabling earlier relapse detection. A promising strategy includes the combination therapy of mTOR inhibitor with taxane-based chemotherapy and the utilization of FDA-approved autophagy inhibitors, such as chloroquine and hydroxychloroquine, to disrupt dormancy-associated survival mechanisms [31]. These approaches offer great potential for preventing recurrence while improving long-term results in patients with residual disease.

## 3. Techniques for MRD Detection in Gynecologic Cancer

**Figure 3:**
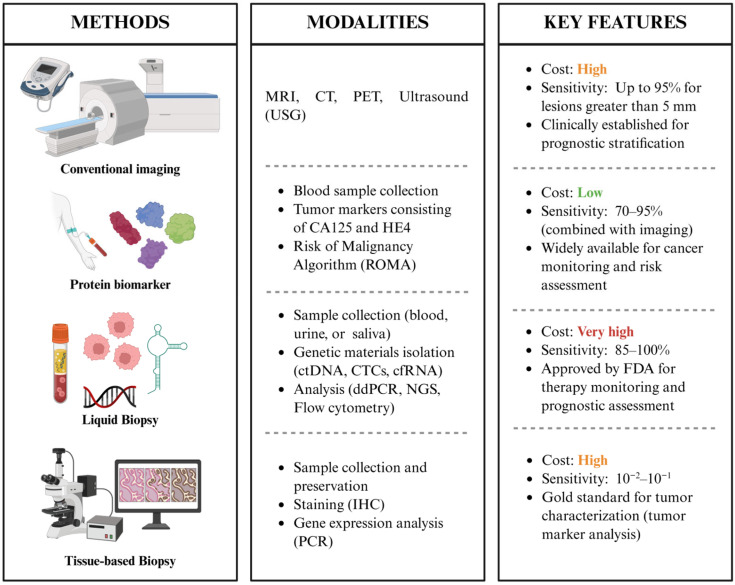
Summary of MRD detection techniques, including their methodological components and clinical features.

The identification of MRD in gynecologic cancers depends on a range of new molecular and imaging technologies aimed at detecting cancer persistence at levels significantly lower than those detected using conventional diagnostic methods. Microscopic residual cells tend not to be precisely detectable through serum biomarkers or standard radiologic evaluations. Consequently, MRD assessment increasingly relies on highly sensitive analytical platforms that can capture tumor-derived signals in blood or tissue. These methodologies, which encompass circulating tumor DNA (ctDNA) assays, circulating tumor cell (CTC) enrichment, next-generation sequencing (NGS), immunohistochemistry, and advanced PCR-based methods, provide complementary insights into tumor burden, genomic alterations, and therapeutic response. Together, they constitute an advanced toolkit that improves risk stratification, allows for earlier detection of recurrences, and assists in the development of more personalized management strategies in gynecologic oncology (Figure 3).

### 3.1. Conventional Imaging Modalities and Serum Biomarkers

Standard imaging modalities, including MRI, CT, USG, and PET, remain essential for evaluating disease progression and detecting residual disease in gynecologic cancers. Among these, MRI exhibits superior performance, particularly with the integration of diffusion-weighted imaging (DWI) and dynamic contrast-enhanced sequences. These approaches enable the differentiation between benign and malignant tissues, minimize background noise, and improve diagnostic accuracy, achieving sensitivity and specificity rates of approximately 90% and 95%, respectively [32]. The combination of PET with CT (PET-CT), particularly utilizing 18F-fluorodeoxyglucose (FDG), offers a functional dimension by revealing metabolic insights associated with higher glucose uptake and glycolytic characteristic in malignant cells, driven by oncogenic pathways including P13K/AKT and MYC [33]. This facilitates the detection of small or concealed lesions that may not be clearly visible with anatomical imaging. Quantitative imaging biomarkers, including standardized uptake values derived and apparent diffusion coefficient provide objective and reproducible metrics related to tumor biology, treatment response, and prognosis [34,35,36]. These biomarkers have shown a correlation with treatment response, prognosis, and the detection of minimal residual disease, thus increasing their significance in precision oncology. Although these modalities improve anatomical staging and indirectly reflect tumor molecular behavior, they often face limitations due to insufficient resolution to identify microscopic residual disease or emerging resistance mutations [37,38].

The evaluation of serum biomarkers, such as cancer antigen 125 (CA125) for ovarian cancer and human epididymis protein 4 (HE4), provides a non-invasive approach for monitoring disease progression and recurrence, often preceding radiographic evidence of relapse [39,40,41]. These circulating proteins reinforce molecular dysregulation within the tumor microenvironment, such as glycoprotein-driven metastasis, EMT induction, and cancer proliferation via MAPK signaling activation [42,43]. While CA125 remains widely used in clinical practice, its sensitivity and specificity vary across cancer type and disease stages, limiting its reliability as a standalone biomarker [44]. HE4 has emerged as a complementary biomarker, particularly in ovarian cancer, where its integration with CA125 in algorithms such ROMA has shown improved diagnostic accuracy. For instance, Samborski et al. reported that combination of CA125 and HE4 improved specificity compared with either marker alone [45]. Moreover, serum biomarker levels enable a quantitative evaluation of tumor burden and biological activity, assisting the qualitative assessment obtained via imaging with diagnostic accuracy approaching 97% [46]. Despite these advantages, both markers predominantly signify downstream products of tumor metabolism and are insufficient to reflect real-time genomic or mutational alterations linked to the survival of residual cancer cells, a challenge shared by conventional imaging modalities. This limitation often results to delays in detecting recurrence or persistent disease, highlighting the need for biologically driven methods, such as liquid biopsy and tumor tissue analysis, which may recognize specific genetic material at subclinical levels, thus offering higher sensitivity and biological specificity for MRD monitoring.

### 3.2. Liquid Biopsy Approaches

#### 3.2.1. Circulating Tumor DNA (ctDNA)

Circulating tumor DNA or ctDNA has emerged as the most advanced and sensitive tool for detecting MRD in solid tumors, providing superior specificity and clinical applicability through a range of advanced technologies. Current approaches involve highly sensitive PCR-based methods, such as digital droplet PCR (ddPCR) and Beads, Emulsion, Amplification, and Magnetics digital PCR (BEAMing), as well as advanced NGS platforms, including whole-exome sequencing and targeted sequencing panels. Each method offers unique advantages in terms of sensitivity, specificity, and multiplexing ability. However, continuous optimization is crucial to improve the analytical functions required for reliable MRD detection, particularly given that ctDNA is often detected at allelic fractions below 0.1% [47].

Accurate quantification of tumor-derived DNA fragments is a pivotal step in MRD monitoring. Methods including digital PCR and quantitative PCR assist in precise measurement of low abundance ctDNA. Advancements in ctDNA extraction techniques, including column-based and magnetic bead-based kits, as well as size-based pre-enrichment strategies, have significantly enhanced analytical sensitivity, thereby enriching tumor-derived fragments for subsequent downstream analysis. In parallel, computational methods employing machine learning have been introduced to improve signal-to-noise ratio, lowering detection threshold for ctDNA and facilitating more robust MRD detection [48,49]. Furthermore, the growing scale and complexity of ctDNA sequencing data requires advanced bioinformatics pipelines. These computational frameworks are designed to effectively identify and quantify rare somatic mutations and other genomic abnormalities while minimizing artifacts, often integrating machine learning algorithms to differentiate real tumor-derived signals from germline variants and sequencing errors [50]. Table 1 showed a comprehensive list of current ctDNA detection methods for gynecologic cancers.

#### 3.2.2. Circulating Tumor Cells (CTCs)

Several enrichment methods, such as immunomagnetic separation and microfluidic devices, have been developed to isolate CTCs from whole blood by applying their unique biological and physical properties compared to normal blood cells [65]. However, the relatively low amount and their dynamic epithelial–mesenchymal transition (EMT) state, which frequently results in the downregulation or loss of epithelial markers, present significant challenges for reliable detection using conventional antibody-based methods. To overcome these limitations, advanced techniques beyond simple antibody-based capture have been explored, including functional assays and single-cell sequencing, which allow more comprehensive characterization of CTCs [66,67]. For example, nanotechnology-based platforms employing metal nanoparticles with unique optoelectronic properties have demonstrated enhanced sensitivity for detecting CTCs at extremely low concentrations, thereby facilitating more accurate analysis within complex biological matrices [68].

**Figure 4:**
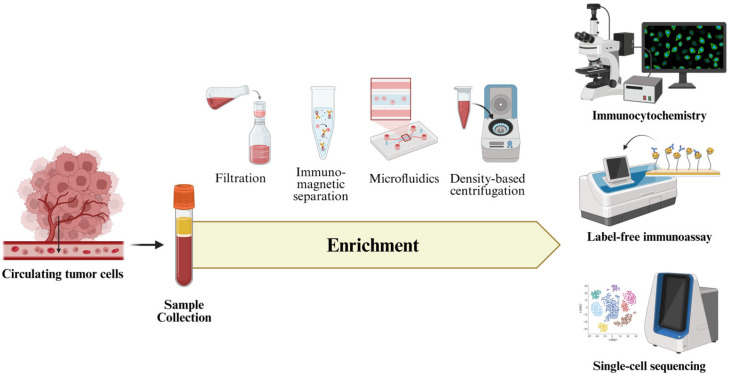
Overview of CTCs analysis, comprising sample collection, enrichment, and downstream molecular analysis. The illustration highlights current enrichment strategies and advanced detection methods.

Furthermore, microfluidic technologies have advanced toward lab-on-chip systems capable of performing CTCs enrichment, detection, and molecular analysis within a single workflow. These platforms enhance efficiency, minimize sample loss, and allow clinically significant analysis. Complementing these developments, immunocytochemistry remains a fundamental technique that provides visual identification and phenotypic characterization of rare CTCs through epithelial markers (cytokeratins, EpCAM) while excluding leukocytes via CD45 negativity [69]. This approach offers molecular detection with morphological validation, allowing the evaluation of cellular integrity, features of EMT such as vimentin and N-cadherin, and the overall CTCs heterogeneity [70]. In addition to these systems, label-free approaches that utilize intrinsic biophysical properties, including cell size, deformability, and electrical impedance, are increasingly recognized for their ability to capture heterogenous CTCs regardless of epithelial marker expression. Additionally, aptamer-based detection methods have recently emerged as a promising alternative, offering high specificity and sensitivity by targeting diverse cell-surface proteins beyond conventional epithelial markers [71]. Figure 4 presented an overview of CTCs analysis, focusing on enrichment methods, detection strategies, and integrated platforms that facilitate comprehensive molecular characterization and clinical application.

Longitudinal surveillance of CTC dynamics could improve risk stratification and inform treatment intensification in ovarian cancer patients [72]. Following surgery or chemotherapy, CTCs have been associated with reduced progression-free and overall survival rates. Furthermore, the presence of CTCs post-treatment in cervical cancer patients has been associated with an increased probability of distant metastasis, emphasizing their potential role in identifying patients who may require more intensive systemic therapies. Concisely, these innovations signify a paradigm shift in CTC analysis, evolving from marker-dependent detection to multifaceted approaches that represent the complete biological complexity of CTC populations.

#### 3.2.3. Exosome and Cell-Free RNA (cfRNA)

Exosomes, essential mediators of intercellular communication, represent abundant sources of multi-analyte biomarkers for cancer diagnosis, such as nucleic acids and marker proteins [73]. The release of cancer-specific exosomes is triggered by oncogene-driven biogenesis and secretion via RAS signaling pathway [74]. However, isolating them from complex biofluids remains challenging due to their nanoscale size, heterogeneous vesicles populations, and the presence of protein aggregates [75]. Conventional ultracentrifugation suffers from low recovery efficiency and high cost, prompting the development of advanced techniques such as immunoaffinity capture, size-exclusion chromatography, and microfluidic platforms to improve exosome purity and yield [76,77]. In parallel, cell-free RNA (cfRNA) analysis is progressively integrated into clinical practices, though its application is limited by low quantity, fragmentation, and degradation. Progress in microfluidics and magnetic bead-based systems now provide automated, high-throughput, and contamination-resistant cfRNA isolation, thereby enabling integration of exosome and cfRNA profiling in liquid biopsy applications [78,79].

For molecular analysis, quantitative polymerase chain reaction (qPCR) remains central for identifying disease-specific transcripts in cfRNA, aiding sensitive surveillance of subtle gene expression changes or residual disease post-treatment [80]. NGS extend this potential by capturing diverse RNA species, including microRNAs (miRNAs) and long non-coding RNAs (lncRNAs), which often enriched within exosomes, thus enhancing both their stability and biomarker potential [81]. Moreover, NGS allows the identification of somatic mutations and copy number variations in cfDNA, providing a more comprehensive genomic view of the tumor and thereby improving MRD testing. Digital PCR (dPCR) further reinforces sensitivity by detecting rare cfRNA and exosomal RNA molecules, offering accurate quantification of MRD prevalences [82]. Complementary to these tools, nanoparticle tracking analysis (NTA) presents direct imaging and quantification of exosome particles, allowing assessment of their size distribution and concentration, which could be associated with disease burden and progression. For instance, molecular profiling of serum-derived extracellular vesicles has proven effective in differentiating ovarian cancer patients based on their treatment responses, indicating their potential as innovative prognostic markers of clinical efficacy. Furthermore, Łukasiewicz et al. reported the importance of cfRNA for early detection and prognostic evaluation in endometrial cancer, employing complex RNA sequencing methods integrated with machine learning algorithm to evaluate tumor-educated platelets and plasma samples [83]. In summary, these advances indicate a systematic advancement in liquid biopsy technologies, enabling more accurate, sensitive, and comprehensive evaluation of exosome- and cfRNA-derived biomarkers for gynecologic cancer diagnostics.

### 3.3. Tissue-Based Approaches

Immunohistochemistry (IHC) and PCR are two complementary tissue-based methods that play a critical role in detecting MRD in gynecologic cancers. Both techniques enable the precise identification of residual tumor cells at concentrations that often fail conventional imaging [84]. IHC is based on antigen–antibody interactions to directly visualize specific protein expression in tissue samples, enabling semi-quantitative evaluation of cellular markers associated with MRD. This approach is necessary for detecting specific protein alterations, including the mismatch repair (MMR) proteins expression, which inform both therapeutic decision-making and prognostic analysis [85]. By localizing and quantifying proteins within the tumor microenvironment, IHC provides valuable insights into cellular pathways and potential drug targets. In endometrial cancer, for instance, IHC is commonly applied to identify mismatch repair deficiency (dMMR), a phenotype presents in approximately 20–30% of cases due to genetic or epigenetic alterations in *MMR* genes [86]. The evaluation typically four key MMR proteins: MLH1, MSH2, MSH6, and PMS2. This serves as a cost-effective alternative to more complex molecular classification strategies, including those proposed by The Cancer Genome Atlas Research Network for endometrial cancer [87]. In contrast, PCR provides highly sensitive and quantitative detection of DNA or RNA sequences, yielding a molecular “fingerprint” of residual disease. This technique excels at detecting mutations, gene amplifications, and fusion transcripts, thereby complementing protein-level information from IHC with detailed genetic insights. For instance, the assessment of microsatellite instability using immunohistochemistry has shown strong concordance with PCR-based approaches in the diagnosis of endometrial cancer, thereby reinforcing its value as a reliable tool in standard clinical practice.

## 4. Clinical Evidence in Gynecologic Malignancies

### 4.1. Ovarian Cancer

Clinical evidence highlighting the importance of detecting MRD in ovarian cancer was reported by Shu et al. (2025), who studied 31 patients with stage II–IV disease that had completed standard treatment (cytoreductive surgery and platinum-based chemotherapy) and achieved complete remission. Prior to treatment, all patients (100%) were MRD-positive. Following treatment, the proportion of patients with detectable MRD decreased to 25.8%, indicating a significant reduction in ctDNA levels. After a median follow-up of 21.4 months, disease recurrence was observed in 15 patients, and those with detectable MRD post-treatment had a significantly higher risk of relapse compared with the MRD-negative group [88]. A similar observation was reported by Zhang et al. (2024), in a study of 51 patients with stage I–III epithelial ovarian cancer [89]. Among the 13 patients who underwent primary debulking surgery followed by adjuvant chemotherapy and remained MRD-positive, 8 (57.1%) experienced recurrence. Furthermore, Weigelt et al. (2017) reported that evaluating circulating cfDNA is essential for detecting reverse mutations in the *BRCA1* and *BRCA2* genes of ovarian cancer patients following therapy. These reverse mutations restore homologous recombination proficiency, thus resulting in acquired resistance to platinum-based chemotherapy and PARP inhibitors [90]. Therefore, cfDNA-based MRD assessment after treatment provides critical information that can directly inform and justify the modification of therapeutic strategies, supporting the clinical necessity to switch to alternative agents when resistance mechanisms are identified, ultimately improving outcomes and personalizing care for ovarian cancer patients.

### 4.2. Endometrial Cancer

In cases of endometrial cancer, Recio et al. (2024) performed ctDNA testing targeting TP53, ARID1A, and PIK3CA in 101 stage I patients, both at initial diagnosis and following surgical intervention [91]. The study found that 58% of patients who were ctDNA-positive at diagnosis, and 52% who remained ctDNA-positive post-surgery, experienced disease recurrence. By contrast, among patients who were ctDNA-negative at diagnosis, only 6% developed recurrence following surgery. Notably, none of the patients who were ctDNA-negative after surgery exhibited any evidence of recurrence (0%). Furthermore, the study demonstrated that ctDNA-positive patients were histologically classified as high–intermediate-risk sarcoma, whereas ctDNA-negative patients were predominantly associated with low-risk histology. These findings suggest that ctDNA testing can provide predictive information not only regarding relapse risk, but also about the underlying histopathological characteristics of endometrial cancer patients [92]. Moreover, Capasso et al. (2025) emphasized that postoperative ctDNA analysis offers significant value for therapeutic management in endometrial cancer. ctDNA information can support decisions throughout maintenance therapy and in recurrent or metastatic disease settings, in addition to serving as a highly sensitive biomarker for early recurrence detection [93].

### 4.3. Cervical Cancer

A multi-center study involving 70 patients with stage IB-IVA cervical cancer was conducted by Han et al. (2023) [94]. HPV ctDNA testing was performed at three time points: immediately after chemoradiation, 4–6 weeks post-chemoradiation, and again at 3 months post-treatment, with patients followed for up to two years. The results demonstrated that individuals with detectable HPV ctDNA at the end of chemoradiation, at 4–6 weeks, and at 3 months post-treatment had significantly lower two-year progression-free survival rates (77% vs. 51%, 82% vs. 15%, and 82% vs. 24%, respectively; *p* < 0.05). Detection of HPV ctDNA reflects the presence of residual cancer cells, as the viral genome commonly integrated within the tumor genome, is released into the circulatory system following therapy-induced cell death. Its persistence or reappearance indicates the presence of viable cancer cells that continue to proliferate and release ctDNA into the bloodstream, thereby proving HPV ctDNA as a highly sensitive marker for minimal residual disease monitoring [95].

MRD assessment in cervical cancer is not limited to HPV ctDNA. Mayadev et al. (2025) conducted a study involving 185 patients with stage IB2-IIB node-positive or IIIA-IVA locally advanced cervical cancer, treated with either chemoradiotherapy plus durvalumab or chemoradiotherapy alone in a 1:1 ratio. Tumor-informed ctDNA analysis was performed at baseline, post-treatment, and three months after therapy. Initially, 98.9% of patients were ctDNA-positive across both groups. Following therapy, ctDNA positivity declined to 39.8% in the chemoradiotherapy group and 35.5% in the durvalumab + chemoradiotherapy group, and by three months further decreased to 36.4% and 23.4%, respectively. This indicated a 13% lower rate of detectable minimal residual disease in the durvalumab + chemoradiotherapy group compared with chemoradiotherapy alone [96]. Importantly, tumor-informed ctDNA is considered more accurate than HPV ctDNA, as it detects a broader spectrum of tumor-specific changes. This broader detection enhances sensitivity and minimizes biases inherent to viral DNA-based assays, thus improving prognostic accuracy and reliability in MRD monitoring for cervical cancer.

In addition to ctDNA, circulating tumor cell (CTC) analysis also plays an important role in the therapeutic management of cervical cancer. Tewari et al. (2020) demonstrated that CTCs, defined as anti-cytokeratin positive and anti-CD45 negative cells, can be isolated from women with advanced-stage cervical cancer. The study found that CTC enumeration served as a useful parameter for monitoring response to bevacizumab therapy; specifically, a reduction in CTC count over the course of treatment was correlated with a decrease in mortality risk [97]. Table 2 summarizes clinical studies demonstrating evidence for MRD detection in gynecologic cancers mentioned in this review.

## 5. Impact of MRD Monitoring on Recurrence Prevention

MRD has emerged as a crucial non-invasive biomarker in the prognosis and management of gynecologic cancers. Its presence is strongly associated with poor outcomes, particularly an increased risk of disease recurrence [98]. In clinical practice, MRD assessment is progressively integrated into decision-making processes to guide adjuvant therapy and determine whether treatment intensity should be increased or reduced post-surgery [99]. The clinical utility of MRD is well documented regardless of some of the evidence coming from studies outside of gynecologic oncology. For example, in a study involving 220 lung cancer patients, Herbst et al. (2025) compared the efficacy of osimertinib, an EGFR inhibitor with placebo [100]. The results suggested an MRD-negative rate of 86% in the osimertinib group, compared to 36% in the placebo group after a period of 36 months. Among patients who discontinued osimertinib, disease-free survival declined from 80 of 112 patients at 12 months to 66 patients at 24 months. This study demonstrates MRD’s potential to identify patients who require continued adjuvant therapy and which do not, in order to prevent disease recurrence.

Beyond its prognostic role, MRD emerges as a promising clinical strategy for early detection of elapse, enabling intervention before residual cells progress to apparent disease. Such early detection has been linked to improved overall survival across multiple cancer types [101]. Additionally, MRD testing supports personalized surveillance programs by tracking tumor-specific genetic and epigenetic changes over time, thereby allowing real-time assessment of therapeutic response. This could additionally assist in directing clinical decisions about the necessity of adjuvant chemotherapy, including the use of immune checkpoint inhibitors.

Growing evidence demonstrates that MRD also provides insight of immune escape and suppression mechanisms. For instance, the presence of MRD in ovarian cancer is associated with the downregulation of CD8^+^ T cells via Interferon (IFN) signaling pathway, indicating immunotherapy resistance. The high expression of PD-L1 has been associated with immune evasion and reduced survival rates [102,103]. Nevertheless, further research suggested that its expression could provide insights into high-grade serous ovarian carcinoma (HGSOC) and endometrial cancer stratifications [104,105,106]. Generally, MRD positivity could signify the overexpression of immune checkpoint pathways or impaired antibody response, attributes which enable tumor cells to bypass immune surveillance and promote recurrence [107,108,109]. These insights underlined the potential of MRD as a potential biomarker for immune response in cancer therapeutic outcomes. In gynecologic oncology, Matulonis et al. (2015) highlighted the clinical significance of this approach by demonstrating a robust correlation between ctDNA-based MRD monitoring of PIK3CA mutations and treatment response in patients with advanced or recurrent endometrial cancer receiving pilaralisib therapy [110]. In summary, MRD offers a powerful framework for personalized treatment and surveillance in gynecologic cancers. This strategy improves recurrence prediction, guides treatment decisions, and facilitates personalized surveillance strategies, thereby enhancing patient outcomes.

Moreover, negative MRD results hold significant clinical relevance, especially following the conclusion of therapy. An MRD-negative status may facilitate decisions to reduce specific treatment components or discontinue therapy, particularly when the current regimen imposes significant symptomatic burdens on the patient. Utilizing MRD to customize treatment intensity may enhance quality of life, decrease treatment burden, and reduce the likelihood of unnecessary toxicity [111]. In summary, MRD offers a powerful framework for personalized treatment and surveillance in gynecologic cancers. This strategy improves recurrence prediction, guides treatment decisions, and facilitates personalized surveillance strategies, thereby enhancing patient outcomes.

## 6. Comparison of MRD in Gynecologic Cancers and Other Solid Tumors

In gynecologic malignancies such as ovarian and endometrial cancer, tumor spread frequently occurs through peritoneal dissemination, ascites, and numerous small peritoneal implants, so residual disease often remains compartmentalized within the peritoneal cavity rather than being solely hematogenous. This means that plasma is not the only relevant sample type for MRD assessment; peritoneal fluid and other local specimens can provide complementary and sometimes more representative information, a situation that is uncommon in most non-gynecologic solid tumors [112]. From a biomarker perspective, MRD testing in ovarian cancer can be integrated with established markers like CA-125, allowing ctDNA to detect recurrence earlier or in cases where CA-125 is non-informative, which gives gynecologic MRD a distinctive multimodal character. In the surgical and pathological context, MRD offers an additional layer of evaluation in diseases where maximal cytoreductive surgery and, in some centers, second-look procedures are performed to confirm the absence of macroscopic disease, enabling direct correlation between operative/pathologic clearance and molecular evidence of residual tumor in a way that is less characteristic of many other solid tumors [113].

## 7. Current Challenges and Limitations in MRD Evaluation

Despite many advantages of MRD assessment, several challenges and limitations both technical and clinical must be considered. In gynecologic cancers, such as endometrial cancer, tumor heterogeneity is a common phenomenon, resulting in considerable genetic and immunohistochemical variation. This heterogeneity may lead to inconsistencies in MRD testing, thus affecting the reliability of the results [114]. Moreover, the quantity of residual tumor DNA present in peripheral blood is often limited in several cases, making technical detection challenging. Under ideal conditions, a 10 mL blood sample yields about 5 mL plasma containing approximately 10 ng/mL DNA. At these concentrations, it is estimated that only about 15 molecules may represent cancer-related ctDNA, a level considered very low and posing significant challenges to the sensitivity and specificity of current MRD assays. When residual disease is present at extremely low concentrations, standard assays may fail to detect it, leading to potentially false-negative results [115]. In addition, MRD assessment raises ethical concerns because sequencing generates a large volume of potentially sensitive personal data, leading to privacy risks; therefore, MRD testing must prioritize robust data protection, genuinely informed consent, and appropriate policies for returning results [114].

Considering MRD clinical applications, sample handling and treatment poses numerous problems that limit the clinical value and widespread adoption of MRD. Primarily, MRD testing requires a considerable amount of liquid biopsy to avoid false-negative results [116,117]. For example, the PCR-based method depends on a minimum blood sample volume of 10 mL [118]. Upon collection, samples must be carefully handled and stored, as delays in processing and fluctuating factors, such as temperature or biosafety level, may compromise sample integrity, hence impacting the reliability of results [119,120]. The absence of universal reference methodologies and varying detection limits in MRD analytical standardization might result in unreliable prognostic value and complicate therapeutic decision-making [121].

Another limitation of MRD testing is its inability to detect the emergence of unrelated second primary cancers in other organs. In other words, MRD is a personalized, tumor-informed mutation assay and is not designed for organ-wide surveillance. Therefore, broader cancer screening requires genetic testing based on hereditary multigene panels and a thorough assessment of family history [122]. Furthermore, MRD testing remains costly and requires advanced technology, creating additional barriers to widespread adoption, especially in developing countries. In many countries, including governmental institutions such as Ministry of Health, current regulations have not established standardized guidance for these methods [123]. For that reason, several strategies have been developed, including the utilization of simplified ctDNA testing via low-cost PCR testing and AI-assisted flow cytometry [124,125]. Improving local capacity through training and raising awareness among healthcare professionals is essential for encouraging the widespread and appropriate implementation of MRD testing [126]. Thus, the establishment of standardized protocols and the execution of robust clinical studies will be essential to reinforce broad and accessible MRD testing for patients with gynecologic cancers.

## 8. The Future of MRD Detection

Building on these insights, this study emphasizes the utility of MRD detection via ctDNA-based assays as a pioneering strategy for prompt and accurate prediction of relapse in gynecologic cancers. The integration of ctDNA-based monitoring, integrated omics analysis, and advanced computational tools support progressive approaches to patient management, with the potential to improve prognostic modeling, optimize surveillance, and tailor therapeutic strategies [127,128]. This combination improves the understanding of cancer progression, accelerates relapse detection, and supports the development of patient-centered treatment plans that address the heterogeneity of tumor biology.

**Figure 5:**
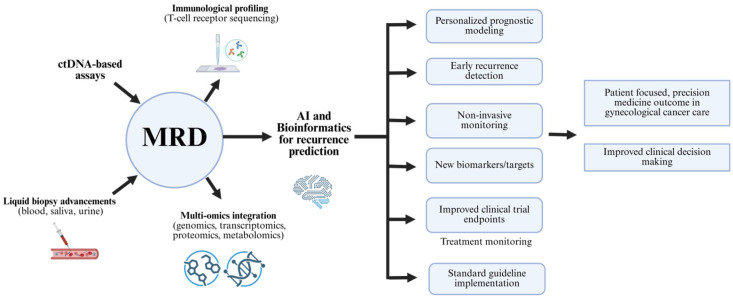
MRD monitoring pathway to support decision-making for subsequent therapy and predict cancer recurrence risk.

Looking forward, the integration of multi-omics and AI-empowered systems is expected to further advance MRD applications by improving risk classification, therapeutic decision-making, and clinical oversight. Broader availability of diagnostic modalities will be essential for translating these innovations into routine clinical practice, thus promoting patient care, optimizing resource allocation, and improving the standard of personalized medicine in gynecologic oncology. Achieving this milestone will require interdisciplinary partnerships among clinicians, computational scientists, and biomedical engineers to harness the opportunities of precision oncology. In the end, these strategies represent a pathway toward individualized, robust, and effective cancer management (Figure 5).

Minimal residual disease has been recognized for its importance in improving precision gynecologic oncology, facilitated by the integration of multi-omics profiling, computational advances, and novel diagnostic platforms. Pairing minimal residual disease monitoring with comprehensive genomic, proteomic, and metabolic analysis, supports deeper profiling of residual malignancy and its biological context, uncovering therapeutic vulnerabilities associated with relapse and drug resistance, and thereby enabling proactive and personalized therapy identification [129]. Parallel advances in machine learning and molecular informatics have further enhanced the accuracy of minimal residual disease diagnostics by leveraging comprehensive molecular and clinical datasets, improving risk classification and supporting personalized treatment decision-making [130,131]. Furthermore, integrating minimal residual disease evaluation with immune characterization, such as T-cell receptor sequencing offers insight into tumor-immune interactions, facilitating earlier detection of relapse related to immune escape [132]. In addition, this approach supports rapid and standardized outcome measurement to accelerate the regulatory approval of effective oncologic therapies. Finally, the convergence of minimal residual disease monitoring with digital health technologies, including telemedicine, wearable biosensors, and cloud-based platforms, enables real-time molecular monitoring beyond large hospitals to smaller clinics and remote communities [133].

## 9. Conclusions

MRD monitoring represents a new approach for detecting recurrence in gynecologic cancers, offering greater sensitivity compared to conventional methods such as imaging and tumor markers. Clinical evidence has shown that MRD status after primary therapy strongly correlates with patient outcomes: survivors with MRD-positive status face a higher risk of recurrence in ovarian, endometrial, and cervical cancers. By implementing cutting-edge technologies, including ctDNA-based liquid biopsy, CTC analysis, and molecular tools using NGS, ddPCR, and single-cell sequencing techniques, MRD enables relapse detection, real-time monitoring of treatment response, and more precise risk classification for adjuvant therapy. Regardless of these benefits, significant challenges remain. Tumor heterogeneity and the limited sensitivity of current methods can result in false negatives when the residual disease burden is minimal. In addition, the lack of standardized protocols, varying interpretation criteria, and high costs of modern technologies like NGS and ddPCR limit their development, especially in resource-limited healthcare settings.

Nevertheless, MRD has demonstrated efficacy as a robust prognostic and predictive biomarker. Its application after therapy reliably provides forward-looking information about recurrent risk and poor survival outcomes, while also serving predictive biomarker for tailoring adjuvant, therapy, or immunotherapy approaches based on residual disease status. Thus, the integration of MRD with multi-omics, artificial intelligence, and progressively non-invasive liquid biopsy strategies has strong potential to accelerate relapse detection and optimize therapeutic decision-making. These advancements can establish MRD as a new clinical endpoint, advance therapy personalization, and strengthen the foundation of precision oncology for gynecologic cancers.

## Figures and Tables

**Table 1:** Current strategies for ctDNA detection and analysis in gynecologic cancers.

Methods	Sensitivity Range (%)	Specificity Range (%)	Applications	Limitations	References
Digital droplet PCR (ddPCR)	75–96%	85–100%	Detection of HPV DNA, mutation analysis, and treatment monitoring.	Limited multiplexing, reliance on predefined targets, and moderate cost-effectiveness.	Kang et al., 2017 [51]; Cheung et al., 2019 [52]; Leung et al., 2021 [53].
Next-Generation Sequencing (NGS)	70–95%	88–98%	Comprehensive profiling, detection of somatic mutations, and identification of molecular subtypes.	High cost, analytical complexity, and substantial infrastructure requirements.	Bolivar et al., 2019 [54]; Lee et al., 2020 [55]; Heo et al., 2024 [56]; Glueck et al., 2025 [57].
Real-time qPCR	24–85%	85–95%	Single-target detection, HPV screening, and basic mutation identification.	Low sensitivity, single-target scope, and limited clinical applications.	Sun et al., 2016 [58]
Multiplex Digital PCR	60–100%	90–98%	Multiple HPV types identifications, simultaneous detection, and screening applications.	Limited validation, high technical complexity, and need for method standardization.	Galati et al., 2022 [59]
Whole Genome Sequencing (WGS)	85–96%	95–100%	Structural variant detection, comprehensive genomic profiling, and personalized diagnostic panel.	Very high cost, complex analytical procedures, and specialized expertise.	Abbas et al., 2021 [60]; Sabatier et al., 2022 [61].
Methylation-based PCR	80–97%	90–95%	Epigenetic markers integration, improved sensitivity, molecular subtype classification.	Technical complexity, lack of method standardization, limited clinical validation.	Elazezy et al., 2021 [62]
AI-enhanced Analysis	84–97.5%	84–95%	Pattern recognition, diagnostic classification, clinical outcome prediction.	Large dataset requirements, reliance on black-box interpretation, clinical validation challenges.	Li et al., 2024 [63]; Paiboonborirak et al., 2025 [64].

**Table 2:** Clinical evidence of MRD implementation in gynecologic cancer.

Study (Year)	Study Design	Biomarker	Sample Size	Statistical Significance
Shu et al. (2025) [88]	Retrospective	ctDNA	Stage II-IV ovarian cancer (*n* = 31)	Median PFS MRD-positive vs. PFS MRD-negative (HR = 6.678, *p* = 0.01)
Zhang et al. (2024) [89]	Prospective	ctDNA	Stage I (*n* = 11), Stage II (*n* = 9), Stage III (*n* = 31) ovarian cancer	Positive MRD post-surgery with relapses as independent prognostic factor (HR = 3.40; 95% CI = 1.02–11.42; *p* = 0.047)
Weigelt et al. (2017) [90]	Prospective	ctDNA	Ovarian cancer (*n* = 19) and breast cancer (*n* = 5) patients with platinum and/or PARP inhibitor resistance (*n* = 19) and	Percentage of ctDNA in breast cancer vs. ovarian cancer was higher (*p* < 0.0005)
Recio et al. (2024) [91]	Prospective	ctDNA	Stage I endometrial cancer (*n* = 101)	Patients with ctDNA positive after surgery at both first time point and longitudinally have an inferior recurrence-free survival (HR = 6.20, *p* = 0.0006 and HR = 15.50, *p* < 0.0001, respectively)
Jamieson et al. (2024) [92]	Cohort	ctDNA	Endometrial cancer (*n* = 24) and Ovarian cancer (*n* = 17), synchronous endometrial (*n* = 2), and endocervical adenocarcinoma (*n* = 1)	Comparison of largest tumor diameter between patients with preoperative ctDNA mutations to patients with no mutations (*p* = 0.075), and advanced (FIGO stage III-IV) disease with *p* < 0.038
Han et al. (2023) [94]	Prospective	HPV ctDNA	Patient with stage IB-IVA cervical cancer treated with CRT (*n* = 70)	PFS of patients with detectable HPV ctDNA vs. undetectable ctDNA; 4–6 post CRT (*p* < 0.03), 3 months post CRT (*p* < 0.001), 2-year post CRT (*p* < 0.0001)
Mayadev et al. (2025) [96]	Randomized controlled trial	ctDNA/cHPV DNA	Adult women with stage IB2-IIB node-poistive or IIIA-IVA any node-status locally advanced cancer (*n* = 185), receive durvalumab + CRT or CRT alone (1:1)	Baseline ctDNA below the median predicted better PFS and OS than higher ctDNA, with HR of 0.61 and 0.55 for durvalumab + CRT and 0.49 and 0.65 for CRT, respectively

## Data Availability

No new data were created or analyzed in this study. Data sharing is not applicable to this article.

## Abbreviations

The following abbreviations are used in this manuscript:

ABC	ATP-Binding Cassette
AI	Artificial Intelligence
ALL	Acute Lymphoblastic Leukemia
AML	Acute Myeloid Leukemia
BCRP	Breast Cancer Resistance Protein
BEAMing	Beads, Emulsion, Amplification, and Magnetics
CA125	Cancer Antigen 125
cfRNA	Cell-Free RNA
CT	Computed Tomography
ctDNA	Circulating Tumor DNA
CTCs	Circulating Tumor Cells
ddPCR	Digital Droplet PCR
dMMR	Deficient Mismatch Repair
dPCR	Digital Polymerase Chain Reaction
DNA	Deoxyribonucleic acid
DWI	Diffusion-Weighted Imaging
EMT	Epithelial–Mesenchymal Transition
EpCAM	Epithelial Cell Adhesion Molecule
ERK1/2	Extracellular Signal-Regulated Kinase 1/2
FDG	Fluorodeoxyglucose
FIGO	International Federation of Gynecology and Obstetrics
HE4	Human Epididymis Protein 4
HPV	Human Papillomavirus
IHC	Immunohistochemistry
IL-6, IL-8	Interleukin-6, Interleukin-8
lncRNAs	Long Non-Coding RNAs
miRNAs	MicroRNAs
MLH1	MutL protein homolog 1
MMR	Mismatch Repair
MPFC	Multiparameter Flow Cytometry
MRD	Minimal Residual Disease
MRI	Magnetic Resonance Imaging
MRP1	Multidrug Resistance Protein 1
MSH2	MutS homolog 2 protein
MSH6	MutS homolog 6 protein
NF-kB	Nuclear Factor Kappa B
NGS	Next-Generation Sequencing
NTA	Nanoparticle Tracking Analysis
PCR	Polymerase Chain Reaction
PD-L1	Programmed Death-Ligand 1
PET	Positron Emission Tomography
PI3K/AKT	Phosphoinositide 3-Kinase/Protein Kinase B Pathway
PMS2	Postmeiotic segregation increased 2 protein
RNA	Ribonucleic acid
ROMA	Risk of Ovarian Malignancy Algorithm
RT-qPCR	Real-Time Quantitative Polymerase Chain Reaction
USG	Ultrasonography
WGS	Whole Genome Sequencing
